# The Clinical Trial Outcomes of Med-Zenith PT-Valve in the Treatment of Patients With Severe Pulmonary Regurgitation

**DOI:** 10.3389/fcvm.2022.887886

**Published:** 2022-06-16

**Authors:** Xiaoke Shang, Nianguo Dong, Changdong Zhang, Yanggan Wang

**Affiliations:** ^1^Department of General Medicine and Geriatrics, Zhongnan Hospital of Wuhan University, Wuhan University, Wuhan, China; ^2^Department of Cardiovascular Surgery, Union Hospital, Tongji Medical College, Huazhong University of Science and Technology, Wuhan, China; ^3^Medical Research Institute of Wuhan University, Wuhan University, Wuhan, China

**Keywords:** transcatheter pulmonary valve, native right ventricular outflow tract, pulmonary regurgitation, Tetralogy of Fallot, coronary artery compression

## Abstract

**Objective:**

Nearly 2/3 of patients with dilated right ventricular outflow tract (RVOT) were excluded from pulmonary valves transplantation due to the lack of size-matched valves. Here, we explored the safety and efficacy of the Med-Zenith PT-Valve for the treatment of patients with severe pulmonary regurgitation.

**Methods:**

22 Patients with severe PR (grade 3+,4+) were enrolled based on the anatomical features of native RVOT and the valve design. The immediate, 3-months and 1-year post-procedural follow-up data were analyzed.

**Results:**

The baseline mean systolic diameters in the distal main pulmonary artery (MPA), MPA sinus junction, MPA sinus, pulmonary annulus, RVOT aneurysm and muscular outlet measured with computed tomography were 33.6 ± 6.1, 34.0 ± 5.8, 37.9 ± 6.0, 32.4 ± 7.3, 41.9 ± 9.3, and 34.4 ± 8.0 mm, respectively. The PT-Valve landing zone was set within these levels. Successful valve implantations were achieved in all patients without noticeable device malposition, coronary artery compression, pulmonary branch obstruction or paravalvular leak during follow-ups. Post-procedural pulmonary artery diastolic pressure increased from 5.8 ± 3.1 to 11.3 ± 2.5 mmHg. In the 3-month and 1-year follow-up, the right ventricular end diastolic volume index reduced from the baseline 181.6 ± 29.0 to 143.7 ± 29.7 ml/m^2^ and 123.4 ± 31.2 ml/m^2^, and the trans-pulmonary valve gradient decreased from 25.6 ± 22.2 to 10.64 ± 3.54 mmHg and 11.16 ± 3.0 mmHg, respectively. The 6-min walk distance increased from 416.6 ± 97.9 to 455.9 ± 64.6 m and 467.8 ± 61.2 m, respectively.

**Conclusion:**

This clinical trial revealed favorable outcomes for the safety, efficacy and feasibility of the Med-Zenith PT-Valve in the treatment of severe PR with significantly enlarged RVOT.

## Introduction

Surgical management of residual pulmonary regurgitation (PR) after initial repair of some congenital heart disease, such as Tetralogy of Fallot (TOF), requires open-heart pulmonary valve replacement with cardiopulmonary bypass. Transcatheter pulmonary valve replacement (TPVR) is a new, less invasive alternative to surgical valve replacement with improved long-term outcome (1–5). However, the current commercially available transcatheter pulmonary valves (TPV) are designed to restore pulmonary valve function in the dysfunctional right ventricle and pulmonary artery (RV-to-PA) conduits (6, 7). To date, the clinical practice of TPV implantation is largely limited to the use of balloon expandable valves, which were only implanted in the native RVOT patients (8). There were reports of several successful implantations of TPV in the native or patched RVOT, with the requirement of landing site diameter less than 29 mm. Because of this limit, about 2/3 of patients with native or patch-expanded dilated RVOT were not suitable for this treatment due to their oversized pulmonary valve annulus. In China, most of the patients underwent surgical reconstruction of the RVOT using a transannular patch technique (9) resulted in a dilated RVOT because the size of annulus usually exceeds the diameter of available percutaneous valves. The need of a device dedicated to the dilated native RVOTs was therefore realized (10–12). However, a large size valve could cause potential coronary artery compression and incomplete expansion of the stent. To prevent the potential risk of coronary artery compression during percutaneous pulmonary valve replacement, 2018 AHA/ACC guideline for the management of adults with congenital heart disease suggested balloon inflation test before transcatheter pulmonary valve placement in the patients with repaired TOF (13). On the other hand, excessive compression or incomplete expansion of the stent could increase the trans-valve gradient, which may affect the durability of the valve and accelerate valve failure (14, 15). To overcome these disadvantages, we reported here the implantations of a novel designed TPV devices (Med-Zenith PT-Valve) and initial outcomes for the treatment of severe PR.

## Methods

### Ethics Statement and Informed Consent

This study was carried out in accordance with relevant guidelines and regulations. Informed consent was obtained from each of the patients or the parents or legal guardians (for patients under 18 years old). Patient's personal information was kept confidentially. The study complied with the Declaration of Helsinki, and obtained the ethical approval from the Clinical Trial Ethics Committee of Huazhong University of Science and Technology at December 8th 2017 ([2017]ID:S310). This trial was registered in China Clinical Trial Registration Center at Oct 26^th^,2017.

Registration number: ChiCTR-OPC-17013126.

http://www.chictr.org.cn/showproj.aspx?proj=22502.

### Patient Selection

The patient enrollment standard and valve selection refer to the Supplemental Materials.

### Device

**Figure 1:**
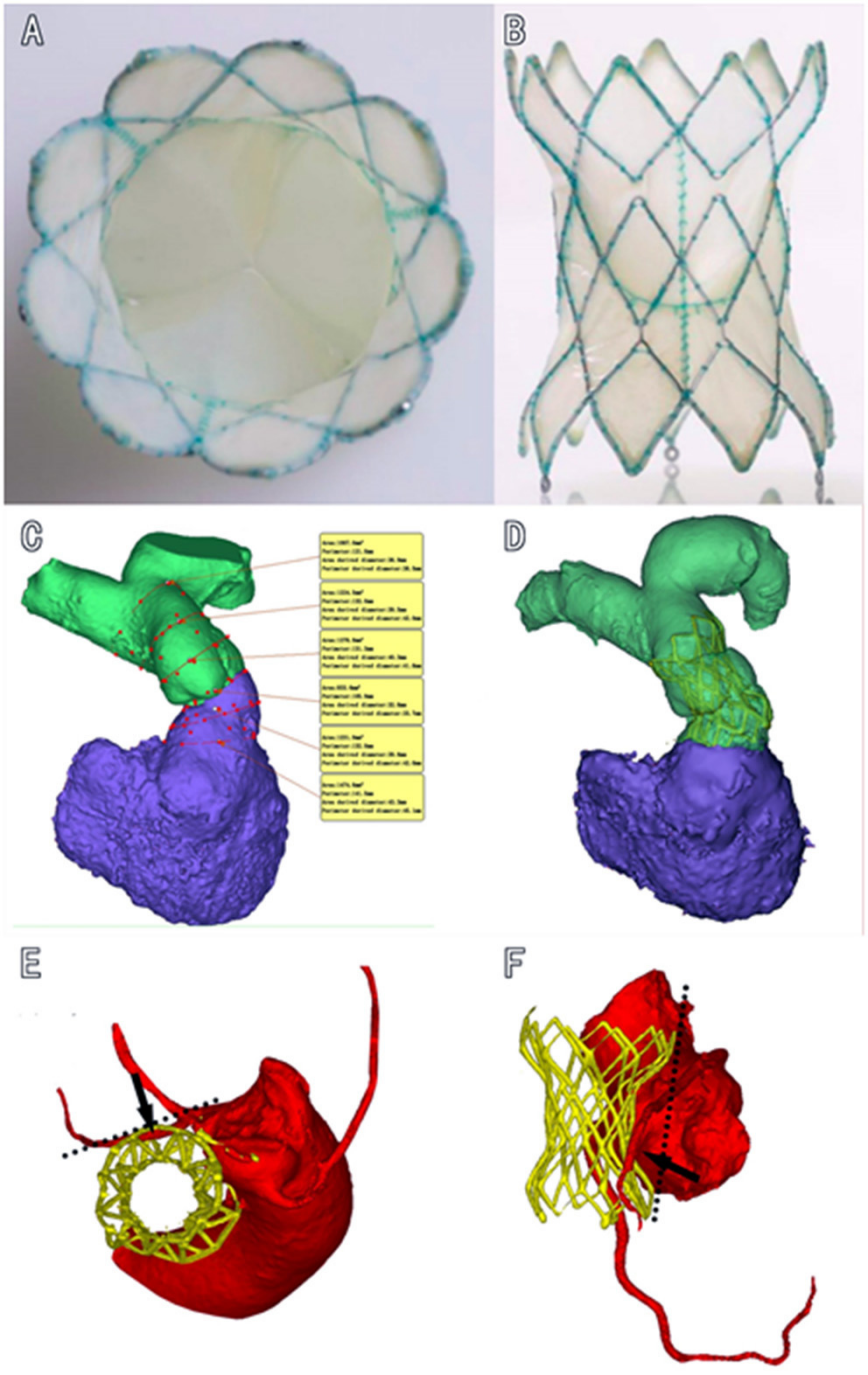
Diagram of the dumbbell-shaped Med-Zenith PT-Valve. **(A)**: Cross section view of PT-Valve. **(B)**: Longitudinal view of PT-Valve; **(C)**: Multi-level measurements based on 3D reconstruction of RVOT and MPA. **(D)**: Valve was implanted into the desired position conformed by post-operational CTA, showing that the dumbbell-shape design of PT-Valve provides sufficient contact surface in the dilated pulmonary artery and RVOT without compression of the valve (waist of the frame remains uncompressed), while the tension force of the device lays on the double ends of the frame. **(E,F)**: Left anterior descending artery is away from the out layer of the stent in the cross-sectional and longitudinal views, respectively. RVOT, right ventricular out flow tract; MPA, main pulmonary artery; CTA, computed tomography angiography.

The Med-Zenith PT-Valve is a porcine pericardial tissue valve mounted on a self-expanding nitinol frame covered by porcine pericardium (Figure 1). The valve frame has five different sizes in order to fit the different morphologies of the RVOT after surgical repair of TOF. The valve frame is made of laser-cut nitinol with a unique symmetrical shape that provides stability and tight seal in the MPA and RVOT to prevent device migration and/or PVL. The outflow and inflow diameters are the same with sizes of 28, 32, 36, 40, and 44 mm, respectively. The length of the frame varies from 38 to 54 mm. The porcine valve diameters in the middle of the frame are 20, 23, and 26 mm, respectively. The diameter of the valve is smaller than the outflow and inflow diameter of the frame to avoid compression. The TPVs are pretreated with specific alcohol and surfactant to mitigate leaflet calcification.

## Results

**Figure 2:**
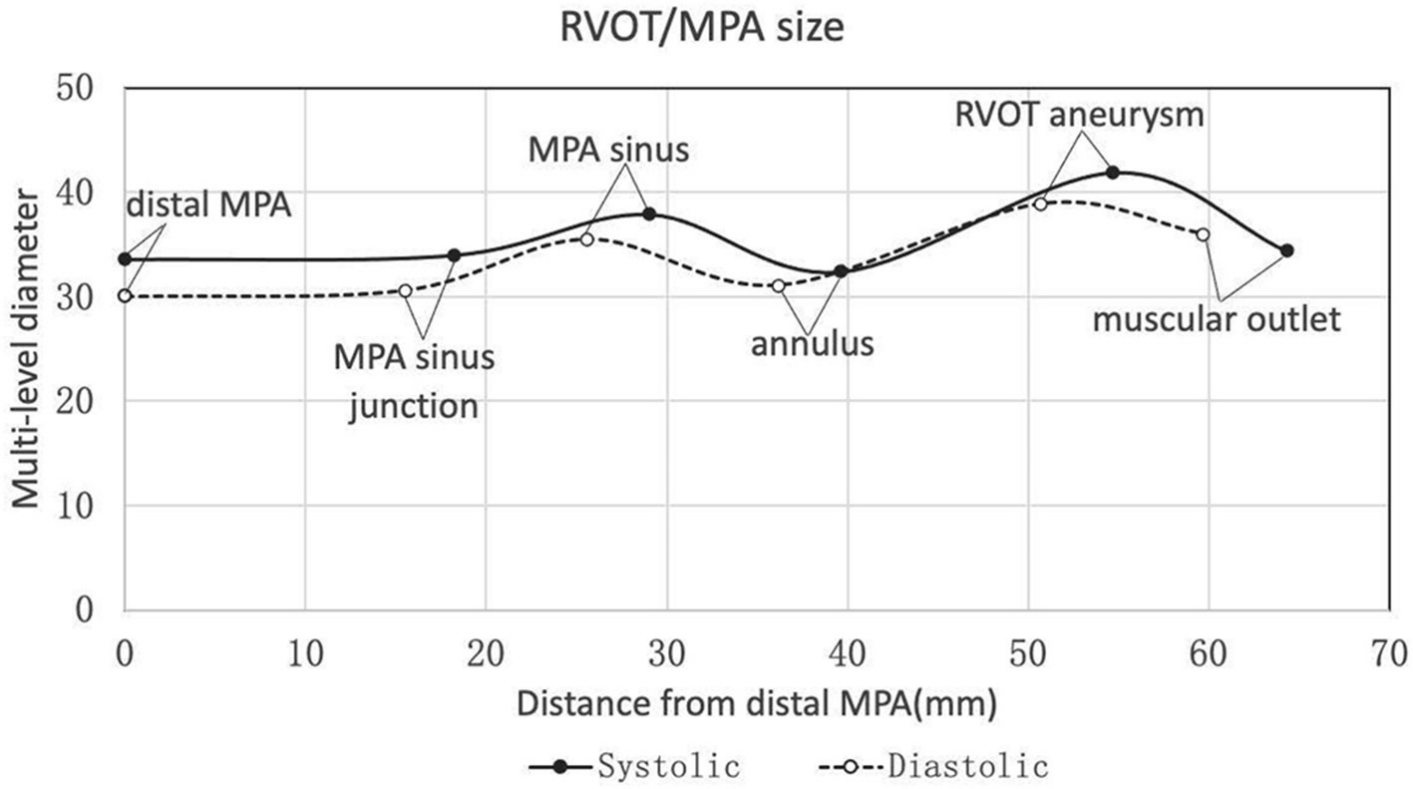
Multi-level diameters and distances of right ventricular outflow tract/pulmonary artery measured by three-dimensional CTA reconstruction in the systole and diastole. CTA, computed tomography angiography.

The baseline characteristics were shown in Table 1. Twenty-two patients were enrolled in this study (17 patients were males). The mean age of the patients was 31.0 ± 9.2 y (weight of 57.9 ± 10.8 kg or 20.7 ± 2.7 kg/m^2^). Heart function was NYHA III-IV for nine patients and NYHA II for eleven patients. Two patients with baseline NYHA class I were enrolled due to their right ventricular end-diastolic volume index (RVEDVI) >160 ml/m^2^, which met the criteria recommended by the 2020 ESC guidelines for the management of adult congenital heart disease. The PR was grade 4+ (severe) for 19/22 patients of which 14 patients also had 3+/4+ tricuspid regurgitation. The mean trans-pulmonary valve gradient measured by echocardiography was 25.6 ± 22.2 mmHg. The mean RVEDVI was 181.6 ± 29.0 ml/m^2^ (measured by cardiac MRI) with RV ejection fraction of 20.3 ± 7.5% and PR fraction of 53.3 ± 13.0%. A large amount of futile circulating in the pulmonary artery aneurysm has been observed in most cases. CTA images showed a substantial variety of RVOT and MPA morphologies with the mean inner diameters (cross section perimeter) at the distal MPA, MPA sinus junction, MPA sinus, pulmonary annulus, RVOT aneurysm and muscular outlet of 33.6 ± 6.1, 34.0 ± 5.8, 37.9 ± 6.0, 32.4 ± 7.3, 41.9 ± 9.3, and 34.4 ± 8.0 mm, respectively (Figure 2, Table 2). The mean distance from the bifurcation to muscular outlet was 64.3 ± 12.4 mm.

**Table 1:** Baseline demographics.

**Baseline Characteristics (*****n*** **= 22)**				
**Male**	**17/22**	**(** ***n*** **)**	**Years between TOF repair and TPVR (year)**	**20.4 ± 8.2**	**(** ***n*** **)**
Age (year)	31.0 ± 9.2		RVOT Type	Native TAP	17
	10–20	2		Conduit	2
	21–30	8		Native Non-TAP	3
	31–40	10	Symptoms	Chest tightness	9
	>40	2		Edema	7
Height (cm)	166.8 ± 9.6			Dyspnea	13
Weight (kg)	57.9 ± 10.8			Palpitation	5
BMI (kg/m^2^)	20.7 ± 2.7		Atrial fibrillation/atrial flutter		7
Diagnosis	PR, rTOF	8	Hypertension		1
	PR, rPA(TOF)	3	Tobacco use		3
	PR, rTOF+rVSD	7	Diabetes		0
	PR, rTOF+TVR	1	Chronic obstructive Pulmonary disease		0
	PR, rTOF+AVR	1	Chronic renal failure		0
	PR, rTOF+RVOTO	2	Stroke		0
rTOF Age (year)	10.6 ± 9.1		Operation time (min)	66.5 ± 16.3	
	<3	5	Radiation time (min)	24.9 ± 8.4	
	3–10	9	Contrast dosage (ml/kg)	2.0 ± 0.7	
	11–20	6	Hospital stay (d)	5.1 ± 1.7	
	>20	2	Valve Size	44–26	7
Surgeries Experienced	1	13		40–26	5
	2	6		36–26	3
	≥3	3		32–23	4
				28–20	3

*BMI, body mass index; PR, pulmonary regurgitation; rTOF, repaired Tetralogy of Fallot; rPA, repaired pulmonary atresia; rVSD, residual ventricular septal defect; TVR, tricuspid valve replacement; AVR, aortic valve replacement; RVOTO, right ventricular outflow tract obstruction; RVOT, right ventricular outflow tract; TPVR, transcatheter pulmonary valve replacement; TAP, trans-annular patch*.

**Table 2:** Multi-plane measurement based on 3D construction of CT.

	**Systolic diameter (mm)**	**Systolic distance from** **bifurcation (mm)**	**Diastolic diameter (mm)**	**Diastolic distance from bifurcation (mm)**
Distal MPA	33.6 ± 6.1	0	30.0 ± 5.6	0
MPA sinus junction	34.0 ± 5.8	18.2 ± 7.3	30.6 ± 5.9	15.5 ± 5.4
MPA sinus	37.9 ± 6.0	29.0 ± 8.5	35.5 ± 6.5	25.6 ± 7.7
Pulmonary annulus	32.4 ± 7.3	39.6 ± 9.7	31.1 ± 7.1	36.2 ± 9.7
RVOT aneurysm	41.9 ± 9.3	54.7 ± 10.3	38.9 ± 7.8	50.7 ± 10.5
Muscular outlet	34.4 ± 8.0	64.3 ± 12.4	36.0 ± 7.0	59.7 ± 14.0

*MPA, main pulmonary artery; RVOT, right ventricular outflow tract*.

Successful valve implantation was achieved in all patients. Each patient was implanted only one device, of which 7/22 patients used the TPVs with size of 44–26 and 5/22 patients used the TPVs with size 40–26. The other TPVs with sizes of 36–26, 32–23 and 28–20 were implanted in 3/22, 4/22 and 3/22 patients, respectively. The mean procedure time was 66.5 ± 16.3 min. No device malposition, coronary compression, or reduced flow to the PA branches occurred during the procedures. After procedure, the pulmonary artery diastolic pressure increased from 5.8 ± 3.1 mmHg to 11.3 ± 2.5 mmHg (*P* < 0.05), while the invasive trans-valvular gradient decreased from 4.1 ± 7.5 mmHg to 2.3 ± 3.3 mmHg (Table 3). No ventricular arrhythmias, valve displacement, regurgitation or PVL above 2+ occurred by after valve deployment. Prophylactic antibiotics were routinely used for 3 days after procedure, and patients were given dual antiplatelet therapy (Aspirin 100 mg/d + Clopidogrel 75mg/d) for 3 months and aspirin (100 mg/d) alone thereafter for 1 year.

**Table 3:** Hemodynamics data in peri-operation.

	**Pre-operation**	**Post-operation**	***t***	***P***
RVP/LVP	0.32 ± 0.08	0.26 ± 0.53	3.682	0.001
PASP (mmHg)	31.1 ± 5.5	32.0 ± 5.5	1.082	0.292
PADP (mmHg)	5.8 ± 3.1	11.3 ± 2.5	6.754	<0.001
mRAP (mmHg)	7.7 ± 3.5	6.1 ± 2.6	2.773	0.011
RVEDP (mmHg)	9.8 ± 3.8	7.5 ± 2.4	3.138	0.005
PA-RV gradient (mmHg)	4.1 ± 7.5	2.3 ± 3.3	1.066	0.298

*RVP, right ventricular pressure; LVP, left ventricular pressure; PASP, pulmonary artery systolic pressure; PADP, pulmonary artery diastolic pressure; mRAP, mean right atrial pressure; RVEDP, right ventricular end diastolic pressure; PA-RV, pulmonary artery-right ventricular*.

**Figure 3:**
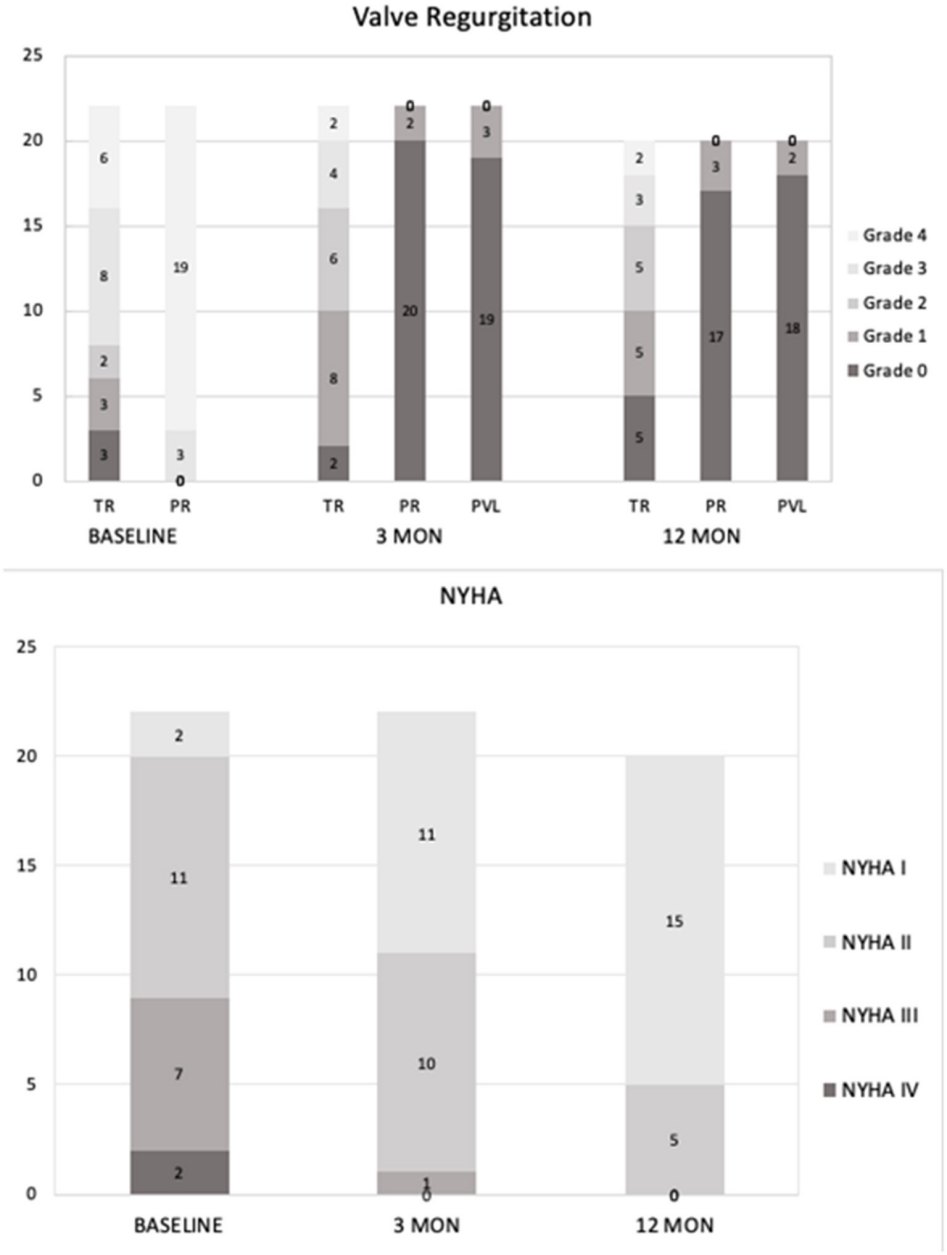
Follow-up data after PT-Valve implantation. Upper panel: Tricuspid regurgitation, pulmonary regurgitation, and transcatheter pulmonary perivalvular leakage at baseline, 3-months and 1-year follow-up. Lower panel: The distributions of NYHA classification at baseline, 3-months and 1-year follow-up.

The patients have been followed up for 13–35 months without early valve decay or reintervention. All patients completed related examinations at the time of 3-month and 1-year follow-up. 2+ valvular or para-valvular regurgitation has not been noted. Five patients were diagnosed with grade 1 PVL or PR without requirement of intervention. Echocardiography and MRI revealed regression of right ventricular remodeling and function, manifested by remarkable reduction of RVEDVI (from 181.6 ± 29.0 to 143.7 ± 29.7 ml/m^2^ at 3 months and 123.4 ± 31.2ml/m^2^ at 1y follow-up, respectively (*p* < 0.05, Table 4). Accordingly, the RV ejection fraction and tricuspid annular plane systolic excursion (TAPSE) were improved (Table 4). Furthermore, valve regurgitation and NYHA class, peak O^2^, 6-min walk distance and NT-proBNP levels were improved continuously after TPVR procedure (Figure 3, Table 4).

**Table 4:** Data of pre-operation, 3-months and 1-year follow-up.

	**Pre-operation**	**3 months**	**1 year**	***t1***	***p1***	***t2***	***p2***
Peak O^2^	14.5 ± 3.8	30.8 ± 9.1	34.3 ± 10.4	9.902	<0.001	8.333	<0.001
6MWD	416.6 ± 97.9	455.9 ± 64.6	467.8 ± 61.2	3.478	0.002	4.370	<0.001
NT-proBNP	1,256 ± 1,415	929 ± 936	805 ± 727	1.243	0.228	1.799	0.088
QRS duration (ms)	114.5 ± 21.4	111.8 ± 16.2	112.4 ± 18.9	0.530	0.602	0.700	0.493
Max gradient (mmHg)	25.6 ± 22.2	10.64 ± 3.54	11.16 ± 3.0	3.351	0.003	2.953	0.008
TAPSE	1.56 ± 0.38	1.68 ± 0.36	1.60 ± 0.36	1.067	0.298	0.405	0.690
RVD	5.27 ± 0.90	4.66 ± 0.86	4.48 ± 0.63	4.205	<0.001	4.966	<0.001
TR velocity	3.30 ± 0.62	3.08 ± 0.47	3.00 ± 0.52	2.348	0.034	2.501	0.031
RAD	5.31 ± 1.13	4.48 ± 0.80	4.49 ± 0.70	5.128	<0.001	5.914	<0.001
RVFAC	32.8 ± 10.2	36.8 ± 10.7	37.3 ± 7.9	1.823	0.083	2.314	0.032
RVEDVI (ml/m^2^)	181.6 ± 29.0	143.7 ± 29.7	123.4 ± 31.2	8.445	<0.001	12.61	<0.001
RVEF (%)	20.3 ± 7.5	31.6 ± 6.6	32.7 ± 4.6	9.429	<0.001	10.59	<0.001
PR fraction (%)	53.3 ± 13.0	1.2 ± 2.4	0.7 ± 1.8	19.03	<0.001	17.59	<0.001

*6MWD, six-minute walk distance; TAPSE, tricuspid annular plane systolic excursion; RVD, Right ventricular diameter; TR, tricuspid regurgitation; RAD, right atrial diameter; RVFAC, right ventricle fraction of area change; RVEDVI, right ventricular end diastolic volume index; RVEF, right ventricular ejection fraction; PR, pulmonary regurgitation. t1, paired t test of pre-operation vs 3-month follow-up; t2, paired t-test of pre-Implant vs. 12-month follow-up*.

One patient had hemoptysis at conduction of Lunderquist guidewire and healed in one day. No other adverse events occurred in the perioperative period, such as tricuspid valve injury, pulmonary artery rupture, pericardial or thoracic hemorrhage, valve displacement and pulmonary branch obstruction. One patient developed infective endocarditis with miliary vegetations in the prosthetic leaflets. It was cured after two months of anti-infective treatment with vancomycin/cephalosporin. No stent rupture, valve thrombosis, embolism or pseudoaneurysm appeared in any cases.

## Discussion

Severe PR after TOF repair is associated with the progressive RV dilation/ dysfunction and subsequent right heart failure. Restoration of pulmonary valve function is therefore important (6). However, the clinical experience with TPVR is largely limited to the dysfunctional RV-PA conduits using the two-balloon expandable TPV devices, including the Melody TPV (Medtronic, Minneapolis, MN, USA) and the SAPIEN Pulmonic THV (Edwards Life Sciences, California, USA) (7, 16). Compared to the RV-PA conduit repair, it is a great challenge to implant a single self-expanding system to fit the wide variety of post-operative RVOT anatomies (13). Although several new TPV devices have been reported in this setting, the experience is very limited (17, 18). Most TOF patients in China were previously operated using transannular patch technique to reconstruct right ventricular outflow tract, which resulted in severe post-surgery PR, MPA/RVOT dilatation or aneurysm and progressive right heart failure. The size of RVOT could exceed the size of currently available TPV devices, which were therefore not suitable for Chinese TOF patients (15). Balloon expanding valves like Edwards Sapiens series have been used to treat pulmonary regurgitation of native RVOT types, but indications are still largely limited by the diameter of annulus (19).

In this study, we introduced a novel TPV device in the treatment of severe PR that occurred after TOF repair, as introduced in our early reports (20, 21). In this cohort with a variety of sizes and morphologies of native RVOT, the immediate, 3-months and one-y outcomes were very satisfactory in terms of efficacy and safety, without showing any serious complications such as death, device migration, PVL, valve malfunction or coronary compression. Excellent valve stability achieved due to the equally expended distal and proximal ends of the stent. In addition, the extended stent length with a well-matched frame provides support to the valve and prevents the potential risk of valve distortion, early progressive malfunction and PVL. More importantly, the unique feature of this device is the symmetric dumbbell-shape design with equal diameters in the inflow and outflow portions of the frame and a progressive incremental in the size of the centrally located inner valve. It is different from any other self-expandable TPV designs, such as the Harmony Valve, Venus-P Valve and Pulsta Valve. The symmetric design of the Med-Zenith PT-Valve provides a sufficient contact surface in the dilated pulmonary artery and RVOT without compression of the centrally located valve. This feature ensures the optional hemodynamics and long-term durability of the leaflet and minimizes the risk of coronary artery compression (Figure 1).

The dumbbell-shaped design of PT-Valve has many advantages. Different from other products, the size of this valve is no longer restrained by the size of annulus but depends on the diameters of the corolla at the two-ends of frame. The currently available Melody and SAPIEN XT/S3 valves have the maximum allowable annular diameter ≤29 mm. Due to this limitation, about 2/3 of patients with native or patch-expanded dilated RVOT have to be excluded from the percutaneous treatment options (10–12). In contrast, the PT-Valve is no longer limited by the diameter of annulus. In fact, most cases in the present study have annulus diameter of 30 mm and above. The PT-Valve significantly extended the valve implantation indication, especially in Chinese patients.

Patients with repaired TOF or abnormal coronary artery anatomy have substantial risk of coronary artery compression during percutaneous pulmonary valve replacement because the left coronary artery usually goes beneath the pulmonary annulus. Coronary artery compression is one of the most serious complications of PPVI (22), which can cause death during operation. To date, several cases of coronary artery occlusion due to compression have been reported in the literature (19). According to current guidelines, in patients with repaired TOF, the trajectories of coronary artery should be determined and the coronary compression test is recommended before percutaneous pulmonary valve replacement (grade Ib) (13). The narrow-waist design in the middle of the PT-Valve leaves no pressure to the annulus and peripheral tissue, thus minimizes the risk of coronary compression. In the present study, our experience demonstrated that with a careful evaluation for the risk of coronary artery compression from the pre-operational CTA while planning the procedure, coronary compression test during the procedure of PT-Valve implantation could often be omitted. Indeed, we only used balloon-inflation coronary artery compression test in 2 patients with RVOT stenosis. Taken together, our results suggest that the unique design of PT-Valve can simplify the procedure of valve implantation and reduce the risk of coronary compression.

Rodriguezgabella et al. (14) reported that excessive compression or incomplete expansion of the stent can increase the trans-valve gradient, resulting in an increased mechanical shear force and asymmetric interaction between the leaflet and the stent, which may affect the durability of the valve and accelerate valve failure. A lowered residual RVOT gradient was associated with the better outcome (15). For the PT-Valve, the valve is in the middle segment of the device, which remains uncompressed after implantation and therefore improves the durability of valves.

Considering the complexity of the native RVOT anatomies, multiple sizes of the frame and valve are required. In the currently available Med-Zenith PT-Valves, there are five different sizes of frames and three different sizes of valves in combination, which makes a flexible selectivity. For instance, with the 26 mm valve in three different frames (36/40/44 mm), we can treatment patients with severely enlarged RVOT.

Our study has confirmed the anatomic complexity of the native RVOT following congenital heart defect (TOF) repair. Rather than a single measurement, we measured diameters (based on 3D reconstruction) of the distal MPA, MPA sinus junction, MPA sinus, pulmonary annulus, RVOT aneurysm and muscular outlet to guide the device size selection (Figure 2, Table 2). 3D printing technique was also used for assisting accurate device size selection if necessary. According to the 2020 ESC guidelines for the management of congenital heart disease, transcatheter pulmonary valve implantation (TPVI) is preferred for patients after TOF repair when anatomically feasible (Ic recommendation). This PT-Valve was specially designed adaptive for the anatomic complexity of the native RVOT, and all patients enrolled for the study were anatomically feasible.

In summary, this clinical trial provided the initial, 3-months and 1-year follow-up outcomes for the safety, efficacy and feasibility of the Med-Zenith PT-Valve in the treatment of severe PR. Our results showed that excellent valve function was maintained without progressive PR or PVL, and regression of right ventricular remodeling has been achieved. Long-term following up in these patients is important to assess persist valve function and durability and stent stability/integrity for the safety and efficacy of this TPV. A following study setup for a CFDA approved trial in China with large-scale in multiple centers in 2020.

### Limitations

The sample size in this study is small due to strict inclusion criteria. Although we have achieved 1-year follow-up outcomes, these patients are still in the follow-up process. In addition, the patients with end-stage heart failure were not included in the study. Thus, whether the conclusions from this study apply to those patients needs further study.

## Data Availability Statement

The original contributions presented in the study are included in the article/Supplementary Material, further inquiries can be directed to the corresponding author.

## Ethics Statement

The studies involving human participants were reviewed and approved by Clinical Trial Ethics Committee of Huazhong University of Science and Technology. The patients/participants provided their written informed consent to participate in this study.

## Disclosure

All authors have read and agreed with the content and submission of the manuscript.

## Author Contributions

XS and ND made substantial contributions to the patient enrollment and surgical procedures. CZ was responsible for data acquisition and analysis. YW supervised the entire integration, data interpretation, and manuscript revision. He is responsible for the overall content. All authors contributed to the article and approved the submitted version.

## Funding

This study was supported by grants from the National Natural Science Foundation of China (NSFC) to YW (Nos. 81420108004, 81270304, 81873507, and 82070348) and grant from the Research Foundation of Commission of Hubei Province to XS (No. WJ2019M173).

## Conflict of Interest

The authors declare that the research was conducted in the absence of any commercial or financial relationships that could be construed as a potential conflict of interest.

## Publisher's Note

All claims expressed in this article are solely those of the authors and do not necessarily represent those of their affiliated organizations, or those of the publisher, the editors and the reviewers. Any product that may be evaluated in this article, or claim that may be made by its manufacturer, is not guaranteed or endorsed by the publisher.
