# Supplementary material for: The Clinical Trial Outcomes of Med-Zenith PT-Valve in the Treatment of Patients With Severe Pulmonary Regurgitation
Source: Front Cardiovasc Med. 2022 Jun 16;9:887886. doi: 10.3389/fcvm.2022.887886 (PMC9243481; doi:10.3389/fcvm.2022.887886)
Supplement: Supplementary file 1 [file Data_Sheet_1.docx]

**Supplemental materials**

**Patient selection**

The study was conducted after approval from the China Food and Drug Administration (CFDA) and Hospital Medical Ethics Committee. From Feb 2018 to Jan 2020, we continuously screened 39 patients who underwent operations for tetralogy of Fallot with moderate to severe pulmonary valve regurgitation diagnosed by ultrasound. 22 patients were considered to be anatomically suitable for PT valve implantation, and the rest 17 patients underwent surgical pulmonary valve replacement due to the lack of suitable TPV device. The reasons for the exclusion of these 17 patients include that the diameter of native right ventricular outflow tract (RVOT) all the way to the main pulmonary artery was ≧40mm, pulmonary hypertension, diffuse dysplasia of pulmonary artery branches, slender right ventricular (RV)-pulmonary artery (PA) valved conduits, coronary artery compressed by pulmonary artery, mechanical tricuspid valve and insufficient femoral vein diameter for introduction of delivery system. All patients had previous surgery for TOF or double ventricular outflow tract (DORV) with trans-annular patch repairment of the RVOT and developed chronic, severe PR with right heart dilation. Multi-module imaging assessment, including transthoracic echocardiograph, computer tomography angiography (CTA) and cardiac magnetic resonance (MRI), were carried out for the assessment of procedure strategy and postoperative evaluation. All data were analyzed in core laboratory.

***Inclusion criteria***

All patients were met the following conditions:

(1) Age ≥ 10 years old or weight > 25kg.

(2) Pulmonary regurgitation fraction ≥ 30% (measured by cardiac magnetic resonance imaging), or pulmonary regurgitation grade 3+/4+ (measured by transthoracic ultrasound).

(3) NYHA class II or above.

(4) Patients with NYHA grade I should meet: right ventricular end diastolic volume index (RVEDVI) measured by cardiac magnetic resonance imaging (MRI) ≥ 150ml/m^2^, or the ratio of right ventricular end diastolic volume (RVEDVI) to left ventricular end diastolic volume (LVEDV) ≥ 2.0.

***Exclusion criteria***

Patients meet any of the following conditions were excluded:

(1) Anatomical evaluation was not suitable for implantation of PT-Valve.

(2) Mean pulmonary artery pressure ≥30mmhg (measured by right heart

catheterization).

(3) Acute heart failure.

(4) History of infective endocarditis.

(5) Allergic to aspirin or heparin.

(6) Other severe diseases or conditions, such as cancer, severe hepatic

or renal dysfunction, acute coronary syndrome, recent or planned major

operation.

(7) Pregnant.

(8) Participating in other clinical trials.

(9) No written informed consent or unavailable follow-up.

**Device**

The Med-Zenith PT-Valve is a porcine pericardial tissue valve mounted on a self-expanding nitinol frame covered by porcine pericardium (Figure 1). The valve frame has five different sizes in order to fit the different morphologies of the RVOT after surgical repair of TOF. The valve frame is made of laser-cut nitinol with a unique symmetrical shape that provides stability and tight seal in the MPA and RVOT to prevent device migration and/or PVL. The outflow and inflow diameters are the same with sizes of 28mm, 32mm, 36mm, 40mm and 44 mm, respectively. The length of the frame varies from 38 to 54mm. The porcine valve diameters in the middle of the frame are 20mm, 23mm and 26mm, respectively. The diameter of the valve is smaller than the outflow and inflow diameter of the frame to avoid compression. The TPVs are pretreated with a specific alcohol and surfactant to mitigate leaflet calcification.

The 21 F delivery system includes an outer sheath in which the valve was compressed, an attachment system that has 3 recessed areas in which 3 corresponding extenders of the frame were placed, and a short, tapered nose cone to facilitate delivery. A loading accessory was developed to facilitate collapse of the valve frame in the outer sheath. The manually controlled release system allows an accurate withdrawal of the outer sheath starting from the distal frame, which controls the expansion of the frame in order to deliver the valve in a precise location. The frame is not repositionable once it has been expanded.

***Valve size selection***

Choosing the size of device mainly depended on the morphology data from the preoperative CTA images and the RVOT angiogram. The criteria for the determination of optimal size were still under investigation. To determine the optimal landing zone, we measured six-level diameters (we named it as Multi-level Measurements based on the 3D reconstruction), including the distal MPA, MPA sinus junction, MPA sinus, pulmonary annulus, RVOT aneurysm and muscular outlet (Figure 2, Table 2). Sometimes, the anatomical morphology, the maximum and/or the minimum diameters of native RVOT/MPA were used to judge the landing level and the landing zone length based on the 3D CTA imaging. The levels of the over-expanded PA sinus and RVOT aneurysm usually represent the maximum diameter of the whole RVOT. The narrowest level was usually the distal MPA-MPA sinus junction or pulmonary annulus. Generally, the chosen valve diameter was 4-8mm larger than the diameter of the anchored area. The corolla diameter needs to exceed the valve ring or the diameter of the pulmonary sinus junction by more than 8mm. If the risk of coronary compression is predicted, the valve diameter can be accordingly reduced. Considering the complexity of pulmonary artery morphology and varying of the MPA length, the distal/proximal landing zone cannot always be located at a particular level. The main anchoring position was the double-end flare, and a reasonable degree of oversize of the flare was chosen based on morphology. 3D printing technology was also used for device sizing if necessary. In the current study, 3D printing model has been used in four cases before implantation.

**Procedure**

All procedures were performed in a hybrid operation room, and the patients were under general anesthesia. Right femoral venous was used for device delivery, while the angiographic catheter was inserted to RVOT (via left femoral vein) to guide precise valve placement. Initial RVOT angiography was performed with steep cranial angulation to outline the RVOT in an optimal view for device implantation. Coronary artery compression test was performed if high risk of coronary compression was predicted by CTA image. Given the unique symmetric design, and other attributes of the device, coronary compression was very unlikely after screening analysis of simulated valve implantation by 3D-Reconstruction. The delivery system was advanced to the pulmonary artery after a Double Curve Lunderquist guidewire (Cook Medical, Bloomington, IN, USA) was placed in the distal left or right lower pulmonary artery. The valve was deployed in the distal main pulmonary artery just beneath the bifurcation, and angiography was performed from the catheter during deployment of the valve frame. The most proximal struct was deployed into the RVOT (Supplemental Figure 1). Once the device was fully deployed in the RVOT, the valve was released from the delivery system with a final unsheathing of the three recessed attachments. The delivery system was then withdrawn carefully to minimize the potential migration or dislodgement of the valve. Pulmonary angiography was then performed to confirm whether the PVL occurred. Hemodynamic assessment was required before and after valve implantation. The venous access was then closed using a Perclose ProGlide device (Abbott Vascular Devices, Redwood City, CA, USA) that has been placed at the beginning of the procedure. The patient was then extubated in the hybrid operating room.

All patients received standardized post-operative care, and any complications were documented. The right ventricular and pulmonary valve functions were determined using echocardiogram and MRI immediately after procedure and at 3-months and 1-year follow-up. The examinations of cardiac ultrasound, CTA and CMR were performed in the core laboratory, and all data were collected by specialists using the MICs # 20.0 (CTA) and circle cardiovascular imaging cvi42 5.1 (CMR) software.


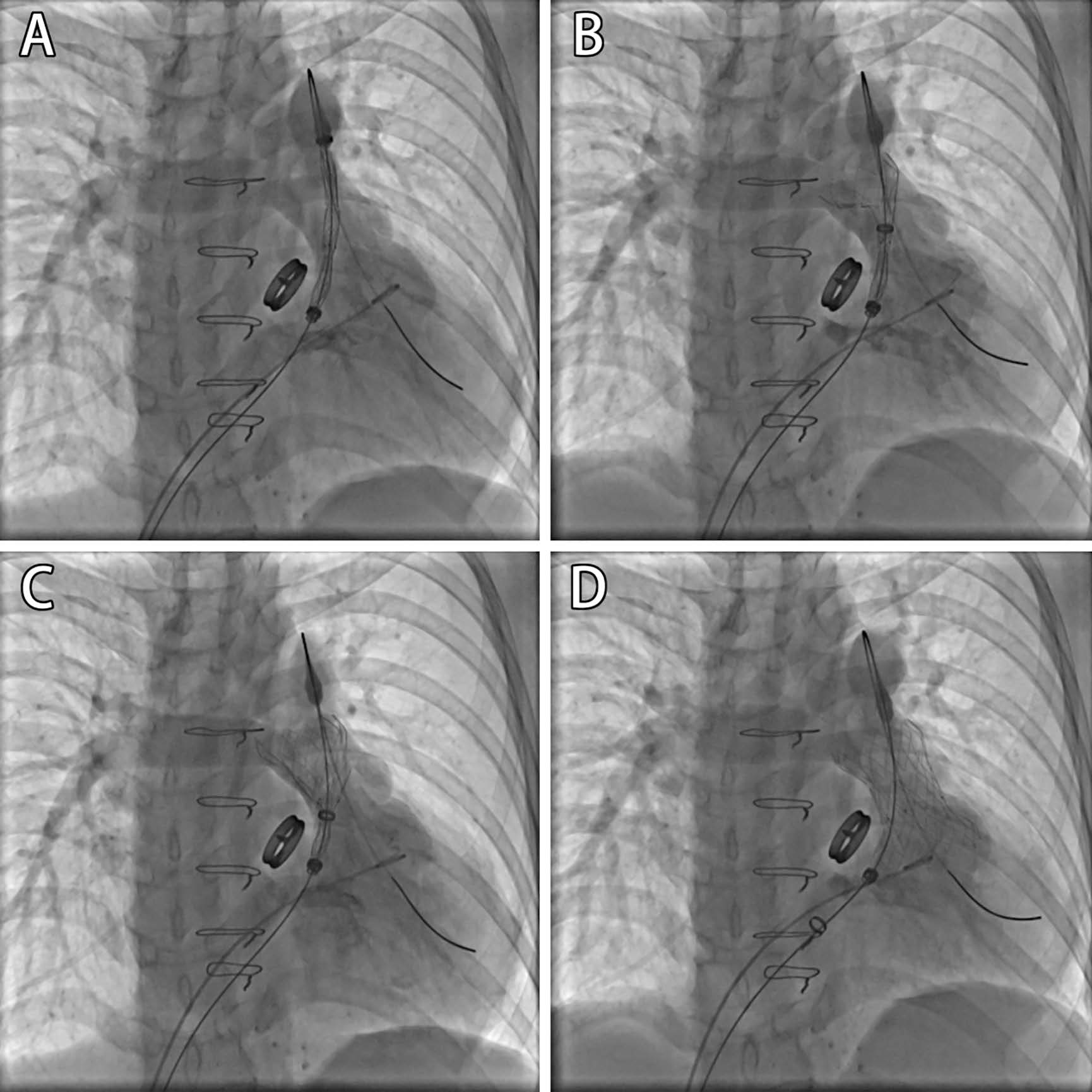


**Supplemental Figure 1**. Procedure of PT-Valve implantation. A: The distal valve-containing capsule was transported to the predetermined position and confirmed under radiography. B: Outflow portions of the device was released. C: Mid-segment of the device was released. D: Inflow portions of the valve was finally released.
